# Isolation, Maintenance and Expansion of Adult Hematopoietic Stem/Progenitor Cells and Leukemic Stem Cells

**DOI:** 10.3390/cancers14071723

**Published:** 2022-03-28

**Authors:** Isabella Maria Mayer, Andrea Hoelbl-Kovacic, Veronika Sexl, Eszter Doma

**Affiliations:** Institute of Pharmacology and Toxicology, University of Veterinary Medicine Vienna, 1210 Vienna, Austria; isabella.mayer@vetmeduni.ac.at (I.M.M.); andrea.hoelbl@vetmeduni.ac.at (A.H.-K.); eszter.doma@vetmeduni.ac.at (E.D.)

**Keywords:** hematopoietic stem cells, leukemic stem cells, ex vivo culture, self-renewal, dormancy, maintenance

## Abstract

**Simple Summary:**

Transplantation of adult hematopoietic stem cells is an important therapeutic tool to help patients suffering from diverse hematological disorders. All types of blood cells can develop from a single hematopoietic stem cell underlining their enormous potential. Intense efforts are ongoing to generate “engraftable” human hematopoietic stem cells to treat hematopoietic diseases and to understand the molecular machinery driving them. Leukemic stem cells represent a low frequency subpopulation of leukemia cells that possess stem cell properties. They can instigate, maintain, and serially propagate leukemia in vivo, while they retain the capacity to differentiate into committed progenitors. Leukemic stem cells are unaffected by many therapeutic strategies and represent the major cause of relapse. We here describe all methods to maintain and expand murine and human hematopoietic cells in culture and describe their specific advantages. These methods are also employed to understand the biology of leukemic stem cells and to identify novel therapeutic strategies.

**Abstract:**

Hematopoietic stem cells (HSCs) are rare, self-renewing cells that perch on top of the hematopoietic tree. The HSCs ensure the constant supply of mature blood cells in a tightly regulated process producing peripheral blood cells. Intense efforts are ongoing to optimize HSC engraftment as therapeutic strategy to treat patients suffering from hematopoietic diseases. Preclinical research paves the way by developing methods to maintain, manipulate and expand HSCs ex vivo to understand their regulation and molecular make-up. The generation of a sufficient number of transplantable HSCs is the Holy Grail for clinical therapy. Leukemia stem cells (LSCs) are characterized by their acquired stem cell characteristics and are responsible for disease initiation, progression, and relapse. We summarize efforts, that have been undertaken to increase the number of long-term (LT)-HSCs and to prevent differentiation towards committed progenitors in ex vivo culture. We provide an overview and compare methods currently available to isolate, maintain and enrich HSC subsets, progenitors and LSCs and discuss their individual advantages and drawbacks.

## 1. The Adult HSC—A Rare, Self-Renewing Cell

**Figure 1:**
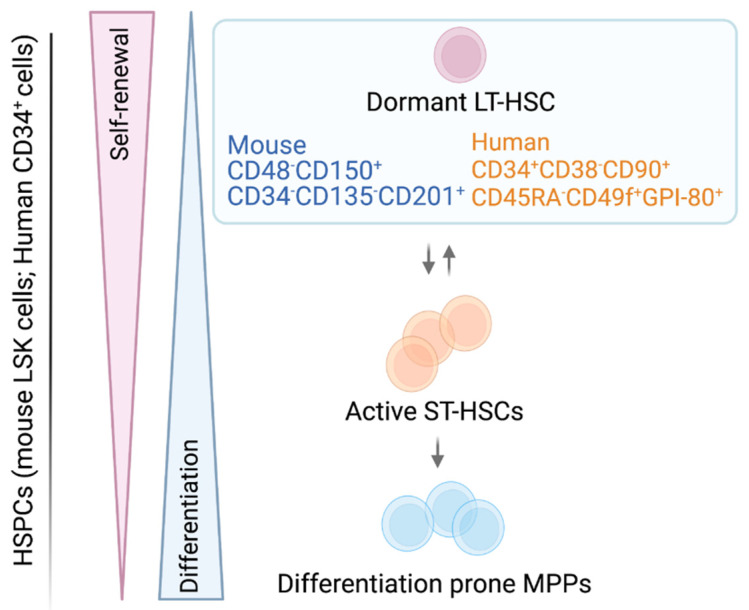
Schematic model of hematopoietic stem and progenitor cells. Surface marker nomenclature for murine/human LT-HSCs cell populations are depicted. The LT-HSCs are activated and transit to ST-HSCs, which in turn gradually commit to more differentiation-prone progenitors. Self-renewal and differentiation are strictly balanced in stem and progenitor cells.

Bone marrow (BM) cell transplantation studies and ex vivo colony formation assays identified the presence of HSCs in the 1950s and 1960s [1,2,3]. Hematopoietic stem cells were functionally defined by their ability to serially engraft transplanted recipients and to replenish all myelolymphoid lineages, through their ability to self-renew and differentiate [4]. These transplantation studies revealed the heterogeneity of the HSC compartment, as HSC subclones were able to repopulate lethally irradiated mice ranged from short term (weeks to months, referred as short term (ST-) HSCs) to long term (more than 6 months, referred as LT-HSCs) [4,5]. Long term HSCs are predominantly in a quiescent/dormant state reflecting their steady-state conditions in the BM [6]. Short term HSCs possess the ability to switch from an active, ready-to-proliferate state back to dormancy [6] (Figure 1). 

Hematopoietic stem cells are rare and represent only 0.005% to 0.01% of all nucleated BM cells. They are isolated based on the expression of a distinct pattern of cell surface markers. Long term HSCs are immune-phenotypically well characterized, while the heterogeneous ST-HSC/(multipotent progenitor)MPP pool lacks a widely used, standardized surface marker expression scheme [7]. Integrating current knowledge, murine HSCs are most commonly first negatively selected for lineage-specific markers (lin^−^), combined with positive selection for cKIT and SCA-1 surface expression by flow cytometry. This procedure results in a cell population called (lin^−^ SCA-1^+^ c-KIT^+^ (LSK)) cells. Within the LSK cell pool, long-term multilineage reconstituting LT-HSCs are highly enriched in the LSK CD48^−^ CD150^+^ CD34^− or low^ CD135^−^ CD201^+^ fraction, while metabolically active ST-HSCs mainly reside in the LSK CD48^− or low^ CD150^−^ CD34^+^ CD135^−^ CD201^−^ fraction. The situation is more complex for the heterogeneous MPPs, which express distinct lineage biases while retaining a degree of plasticity. Different combinations of surface marker expressions and naming strategies exist (LSK CD48^− or +^ CD150^− or +^ CD34^+^ CD135^− or +^ CD201^−^) (Figure 1) [6,8,9,10,11,12,13,14,15,16,17] (Table 1). Further dissection and definition of the ST-HSC/MPP pool, aiming to standardize immune-phenotypic and functional characteristic of the distinct MPP populations were recently reviewed by Challen et al. [7].

In human BM and cord blood (CB), a lin^−^ CD49f^+^ CD90^+^ CD45RA^−^ CD34^+^ CD38^−^ CD133^+^ CD201^+^ GPI-80^+^ surface profile is most commonly used to define LT-HSCs with multilineage reconstitution potential, while an universal definition for human MPPs is still lacking [18,19,20,21,22,23,24] (Table 1, Figure 1). 

With the technological breakthrough of single-cell transcriptomics, it has become evident that predefined flow sorted stem and progenitor populations are only snapshots of HSC differentiation, which is rather a continuous process consisting of metastable cell states. Individual HSCs gradually acquire linage biases without passing through discrete hierarchically organized progenitor populations, suggesting that there is no obvious boundary between LT-, ST-HSCs and MPPs [25,26,27].

**Table 1:** Cell surface markers of murine or human LT- and ST-HSCs and progenitors; some markers can be used for both species.

HSC Surface Markers					
Murine		Refs.	Human		Refs.
CD19, CD45R CD11b, Ly-6G CD3	Lineage negative selection (Lin^−^)	[28,29,30]	CD45RA	CD45 isoform with specific molecular weight	[31]
CD117	Type III transmembrane tyrosine kinase receptor (c-KIT)	[29,32,33,34]	CD38	Cyclic ADP ribose hydrolase	[31,35]
SCA-1	Lymphocyte activation protein-6A (Ly-6A/E)	[29,30,32,33]	CD49f	Integrin α-6	[20,31]
CD48, CD150	Signaling lymphocyte activation molecule (SLAM) family protein	[9,11,12,16]	CD90	Thy1	[30,31,36]
CD34	Transmembrane phosphor-glycoprotein	[9,16,33,37]	CD34	Transmembrane phosphor-glycoprotein	[29,36,38,39]
CD201	Endothelial protein C receptor (EPCR)	[40,41]	CD201	Endothelial protein C receptor (EPCR)	[41,42]
CD135	Fms-like tyrosine kinase 3 receptor (FLT3-R); FLK2	[9,16,33]	CD133	AC133, Prominin-1	[43,44]
			GPI-80	Glycosylphosphatidyl Inositol-Anchored Protein GPI-80	[21,23]

## 2. Leukemic Stem Cells (LSCs)—Villain to Its Heathy Counterpart

Certain leukemias are hierarchically organized with LSCs being on the top, analogous to the hematopoietic tree. Like HSCs, LSCs possess typical stem cell characteristics including the ability for dormancy or self-renewal [45]. The LSCs are responsible for the initiation, progression and relapse of acute myeloid leukemia (AML) or chronic myeloid leukemia (CML) [45,46,47]. Their disease-initiating capacity term them also ‘leukemia-initiating cells’ (LICs). In AML and CML, LSCs can switch back to a quiescent state to evade chemotherapeutics [46,48,49,50]. This leaves LSCs frequently unaffected by therapeutic strategies and may cause relapse [51,52,53].

In AML, in vivo leukemia initiation studies are the gold-standard functional assays to define LSCs. Leukemia development directly correlates with the number of AML-LSCs in the primary sample and predicts clinical outcome [54,55,56]. This functional criterion is not shared by CML-LSCs, as a vast majority of primary samples from chronic phase (CP)-CML patients do not engraft in immunocompromised mice. This may be related to the lower mutational burden in CP-CML, compared to blast phase (BP)-CML, which mirrors an acute leukemia [57,58,59]. 

The LSCs predominantly belong to the CD34^+^ CD38^−^ compartment [46,47,60,61]. The availability of severely immunocompromised mouse strains unveiled the potential of leukemia initiation of AML-LSCs. The LICs are not restricted to the CD34^+^ CD38^−^ cells but are also found in the CD34^−^ fractions [62,63]. Transcriptome analysis and studies of the differentiation capacities of CD34^+^ and CD34^−^ AML LSCs revealed that progenitor as well as mature cells may serve as the origin of LSCs, through an acquired ability to self-renew [64,65,66,67]. In this respect, the more advanced stage of BP-CML mirrors an AML, in which LSC activity is found within CD34^+^ and CD34^−^ populations [64,68,69]. In contrast, LSCs in CP-CML arise from cells with high inherent self-renewal, such as CD34^+^ CD38^−^ HSCs, as the driver mutation (BCR–ABL1) impairs self-renewal [70]. Besides CD34 and CD38, a growing list of AML and CML LSC-selective cell surface markers were identified, enabling classification of LSCs (Table 2). 

## 3. BM Niche of LT/ST-HSCs and LSCs

The LT/ST-HSCs reside in specialized niches that in the cavities of the trabecular regions in long bones. Multiple cellular types, soluble factors and components of the extracellular matrix form these niches [90,91]. The endosteal niche, located at the endosteal surface of the bone is characterized by bone regenerating osteoblasts (OBs) [92]. The OB cells regulate LT-HSCs quiescence by producing CXC motif chemokine 12 ligand (CXCL12), transforming growth factor β (TGFβ) and angiopoitin-1 (ANG-1) [90]. The arteriolar niche is located aside vascular structures and brings LT/ST-HSCs in close proximity to endothelial cells (ECs) and mesenchymal stromal/stem cells (MSCs) [93]. The ECs regulate dormancy and self-renewal via cell-cell contacts (e.g., via E-selectin) and by expression of stem cell factor (SCF), CXCL12 and Notch ligands [11,90,94,95,96]. The MSCs play a dominant part and are subdivided into Leptin^+^, CXCL12 abundant reticular (CAR) or Nestin^+^ cells, which provide soluble factors for HSC maintenance including SCF, thrombopoietin (TPO), CXCL12, fibroblast growth factor 2 (FGF-2) and WNT ligands [90,92,94,97,98,99]. The peripheral sympathetic nerves, ensheathed by non-myelinating Schwann cells (NMSCs), constitute another component of the arteriolar niche. Circadian adrenergic signals from nerve terminals regulate the production of CXCL12 in Nestin^+^ MSCs. Approximately 20% of HSCs are in direct contact with NMSCs, which are maintaining LT-HSC quiescence by producing TGF-β [100]. When activated, ST-HSCs relocate to the Leptin^+^ MSCs containing perisinusoidal area [100]. Adipocytes, pericytes, fibroblasts, macrophages and megakaryocytes are also part of the BM niche and modulate functions of HSCs [90]. 

The LT/ST-HSCs and niche cells can interact through juxtacrine signaling (or contact-dependent signaling) via N-cadherin, vascular cell adhesion molecule 1 (VCAM1)/very late antigen-4 (VLA-4), cKIT/membrane bound SCF or NOTCH receptor/ligand [101,102,103]. Niche cells provide extracellular matrix (ECM) proteins including glycoproteins (e.g., fibronectin), glycosaminoglycans (e.g., hyaluronic acid), collagen IV and matrix remodeling enzymes. The LT/ST-HSCs bind ECM proteins mainly via integrins, which triggers intracellular signals. Of note, the extent of signaling may be modulated by the “stiffness” of the surrounding via mechanotransduction. Stiffness, composition and location of the niche are crucial to modulate HSC behavior [104,105,106,107]. Besides signaling through integrins, ECM embeds LT/ST-HSCs in their niche and provides a reservoir for soluble factors. These factors and cell-matrix interactions impact the balance between stemness and differentiation [97].

In AML and CML, the BM is tightly packed with malignant hematopoietic cells causing disturbed niche structures and hematopoiesis [108,109]. The leukemic blasts occupy and rearrange the BM niches to establish self-protective niches. This process is regulated by stromal-secreted chemokines, CXCL12 and the CXCR4 receptor and creates a “reduced” version of the conventional BM niche [110]. It impairs normal homeostasis and promotes disease progression [45,109,111]. In this protective environment LICs/LSCs are less amenable to chemotherapeutics [45,73,108,112]. Attempts to mimic the BM niche in vitro by 2-3 D techniques try to reduce the transitional gap between in vitro and in vivo research [113,114].

## 4. Essential Factors for HSC Quiescence and Self-Renewal—Cell Cycle Components as Mediators

The LT/ST-HSCs are capable of self-renewal or differentiation. Under homeostatic conditions, most LT-HSCs exist in the G_0_ phase of the cell cycle and are considered “quiescent”. Signals from the BM preserve the quiescent state and protect from cell damage and exhaustion [115]. Quiescent HSCs are fundamental for transplantation and provide the potential for long-term engraftment [116]. 

Symmetric cell division results in two identical daughter cells, that either keep stem cell properties or differentiate. During asymmetric cell division, one daughter cell preserves stem cell characteristics, while the other one undergoes differentiation. During asymmetric division, cell fate determinants are unequally distributed, like tyrosine-protein kinase receptor 2 (TIE2) or NOTCH1. Similarly, active mitochondria, lysosomes and autophagosomes are unevenly shared. This procedure gives rise to a metabolically active, differentiated and short-lived progenitor cell and a metabolically less active, undifferentiated and long-lived HSC progeny [117,118,119,120]. 

Cell cycle regulators are critical factors in maintaining HSC quiescence and re-inducing proliferation. The cyclin C/cyclin dependent kinase 3 (CDK3) complex regulates G_0_ phase, while G_1_ is controlled by cyclin D/CDK4 and CDK6 complexes [121,122]. The balance of CDKs and cyclin dependent kinase inhibitors (CDKIs) control the transition from G_0_ to G_1_. 

The G_1_ -specific CDKIs comprise of two families. The CIP/KIP family consists of p21 (p21^CIP^, CDKN1A), p27 (p27^KIP^, CDKN1B) and p57 (p57^KIP2^, CDKN1C). The second CDKI family includes four INK4 members: p15, p16, p18 and p19 (CDKN2A-D). The INK4 family members bind to CDK4/CDK6 and inhibit their kinase activities by interfering with their association with D-type cyclins. In contrast, the CIP/KIP family members bind and inhibits both cyclin and CDK subunits [121,123]. In the absence of CDK4/6 kinase activity, retinoblastoma (RB) proteins (RB, p107, p130) remain under-phosphorylated. Thus, they bind to E2F transcription factors and prevent them from being active. Entry into S phase and cell cycle progression are inhibited [121]. Among all CDKIs, p57 has the highest expression in LT-HSCs. Members p57 and p27 maintain HSC quiescence by preventing nuclear translocation of HSC70/cyclin D1 and consequent activation of CDK4/6 [124]. From the INK4 family, p15 and p18 have been found to negatively regulate HSC ex vivo expansion [125,126,127]. During genotoxic stress states and aging, p19 preserves HSCs in a quiescent state protecting them from apoptosis [128].

**Figure 2:**
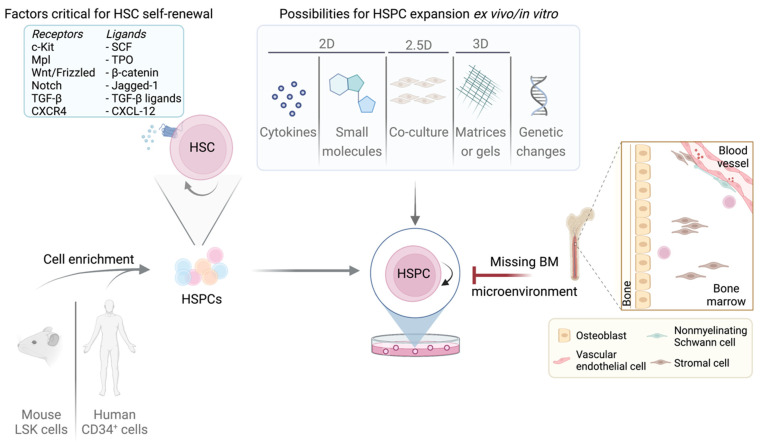
Overview of murine/human LT/ST-HSCs expansion possibilities and limitations. Self-renewal in LT/ST-HSCs is strictly regulated by multiple factors. The most important receptors and their corresponding ligands are listed. Purified LT/ST-HSCs can be cultured by various methods summarized in the box. Adult LT-HSCs reside in the BM niche which is absent upon cultivation, presenting the greatest limitation and challenge.

Pathways triggered by extracellular signals frequently converge on cell cycle regulators (cyclins, CDKs, CDKIs, transcription factors, microRNA, etc.) [115,129,130]. The SCF/c-KIT, TPO/c-MPL and NOTCH ligands/NOTCH1-4 signaling support HSC survival, self-renewal and regeneration [131,132,133,134]. The CXCL12/CXCR4, TGF-β/TGFβR and ANG-1/TIE1/2 signaling is essential to maintain HSCs in a quiescent state [100,135,136,137] (Figure 2). 

Stimulation of c-KIT and MPL activates signal transducers and activators of transcription (STAT), mitogen activated protein kinase (MAPK) and phosphoinositide 3-kinases (PI3K)/Akt pathways to enhance HSC survival and expansion ex vivo [138,139,140,141,142,143,144,145]. The TPO drives the expression of two negative cell-cycle regulators (p57, p19) and the transcription factor homeobox B4 (HOXB4), a potent promoter of LT-HSC self-renewal [146,147].

Activation by STAT5 typically drives cell survival and proliferation but may also mediate quiescence through driving the expression of TIE2, p21 and p57 [148,149]. The hypoxia-inducible factor 2 alpha (*HIF2α*) is a direct STAT5 target gene, which upregulates *c-MYC*, *VEGF* and glucose metabolism. Under hypoxia, STAT5 can impose a long-term proliferative advantage on the CD34^+^/CD38^−^ HSC population, but not on progenitors [150]. Thus, depending on niche signals, STAT5 links self-renewal to a quiescence-typical metabolic profile and cell cycle arrest.

NOTCH signaling promotes LT-HSC self-renewal and inhibits differentiation [95,151]. Unlike secreted niche factors, NOTCH signaling is a juxtacrine communication pathway between NOTCH ligands (Dll1, Dll4, Jagged1, or Jagged2) and NOTCH receptors (NOTCH1-4) expressing cells. Ligand-bound NOTCH receptor is cleaved, releasing the NOTCH intracellular domain, which translocates to the nucleus and alters gene transcription. NOTCH signaling provides direct transcriptional downregulation of p57 and upregulation of several genes that are important for HSC activation, including *HES1*, *GATA2*, *cMYC* [152,153,154,155]. 

Many important signaling pathways, such as WNT/beta-catenin, MAPK, PI3K/AKT/GSK-3 and JAK/STAT regulate expression of c-MYC. As a transcription factor, c-MYC antagonizes p21 and p27 activity by inducing the expression of D-type cyclins, thus enabling the formation of cyclin D-CDK4/6 complexes [156,157]. In fact, WNT signaling has been mostly characterized in dividing cells. However, LT-HSC appear to have the highest percentage of WNT activity, whereas MPPs have the lowest. This indicates that WNT signaling enforces quiescence, probably by upregulation of p21 [158,159].

The CXCL12 (also termed stromal cell-derived factor 1, SDF1) has pleiotropic effects. It is not only a chemoattractant for HSC homing, but also a regulator of HSC quiescence as it upregulates p57 and limits generation of reactive oxygen species (ROS) and genotoxic stress [136,160]. Similarly, TGF-β potently inhibits HSC proliferation, regulates quiescence, and protects HSCs from excessive differentiation signals. It does so by downregulating cyclin D2 and upregulating p15, p21, p27 and p57 [125,137,161,162,163,164,165]. The P53 is highly expressed in LT-HSCs. It regulates quiescence by inducing p21 expression and driving the expression of the transcriptional repressors GfI-1 and Necdin [166,167]. Necdin directly inhibits E2F1, while GFI-1 decreases the expression of inhibitor of DNA binding and differentiation-2 (ID2), an inhibitor of RB and repressor of CDKI p21 and p27 [168,169,170]. Upon activation, p53 is repressed by the ETS family transcription factor, MEF/ELF4, enabling entry into the cell cycle [171].

Among other transcriptional regulators, MEF/ELF4 and CDK6 regulate the exit from dormancy, while PBX-1 and EVI-1 maintain LT-HSC self-renewal [171,172,173,174,175].

Abnormal activation of HSC signaling pathways induce cell cycling, exhaustion or development of leukemia [176]. Hematopoietic challenges such as inflammation, BM transplantation or oncogenic transformation also trigger activation and proliferation of LT/ST-HSCs [177,178].

## 5. Signaling and Metabolic Changes in LSCs

Malignant transformation is caused by genetic or epigenetic alterations due to hereditary or environmental conditions. The fusion protein BCR–ABL1, a constitutively active tyrosine kinase, is the unique hallmark and main driver of CML leukemic cells and LSCs, as it is present in >90% of the patients [179]. Both BP-CML and AML are genetically more complex and heterogeneous with subgroups showing individual mutations. Early mutations improve the potential to self-renew and may impair differentiation leading to heterogeneously expanded clones of pre-leukemic HSCs in patients [180,181,182]. Late co-expressing mutations occur in signaling pathways (e.g., FLT3), promote proliferation and enhance block of differentiation [183,184]. The molecular defects underlying AML are complex with at least 24 different genetically defined subtypes [185]. Expression of AML-associated mutations and fusion genes involve transcription factors or epigenetic regulators, such as DNMT3A, IDH1/2 and TET2 mutations or MLL-, NUP98- and AML1-fusions to name few. Co-expression of mutations in tyrosine kinases and other signaling mediators, such as FLT3- N/K-RAS-, KIT- mutations support proliferation leading to a more aggressive disease progression [186]. 

Common mRNA and epigenetic signatures are found in AML LSCs irrespective of the oncogenic driver or immunophenotype. Ng and colleagues identified a panel of 17 genes (called LSC17), which are highly expressed in LSCs (relative to the bulk of AML cells). High LSC17 expression reflects stemness properties of LSCs and resistance to standard AML therapy. The LSC signature genes include *GPR56, AKR1C3, CD34, EMP1, SMIM24, SOCS2, CPXM1, CDK6, KIAA0125, DPYSL3, MMRN1, LAPTM4B, ARHGAP22, NYNRIN, ZBTB46, DNMT3B* and are predictive and/or prognostic biomarkers [187,188].

Sachs et al. demonstrated that transcriptional profiles of self-renewal and proliferation are distinct in AML LSCs. LSC-specific self-renewal signature (*CD69*, *S100A4*, *MYB, ADA*, *MRI1*, *CKS2*) and proliferation genes (*H2AFZ*, *BCL2A1D*, *CD36*) were identified based on high expression in AML LSCs relative to normal hematopoietic stem/progenitor cells (HSPC)s. Using cell surface markers, CD69 and CD36 allowed the isolation of different subsets of LSCs. The CD69^high^ LSCs were capable of self-renewal and poorly proliferative, whereas the CD36^high^ LSCs did not inflict leukemia and were highly proliferative [89]. These genes were not found as a signature in normal HSPCs and may represent a unifying feature for the identification of LSCs [62,89,187,189]. 

The HSCs rely on glycolysis in the hypoxic BM microenvironment, rather than oxidative phosphorylation (OXPHOS) [190]. In contrast, AML and CML LSCs have higher mitochondrial mass and an increased oxygen consumption rate with a greater dependency on mitochondrial function and OXPHOS [191]. Mitochondrial respiration generates high levels of ROS in bulk CML and AML blasts relative to LT/ST-HSCs [192,193]. High ROS levels can induce oxidative DNA damage, high mutational burden and genomic instability, which may impair stem cell function [194,195,196,197]. Quiescent AML LSCs generally have low ROS levels compared to cycling LSCs and bulk AML cells [198]. These cells may revert to glycolysis or using mitophagy to reduce their dependency on mitochondrial respiration [199,200]. Quiescent low-ROS AML LSCs frequently overexpress BCL-2 and are dependent on amino acid uptake and OXPHOS [198,200]. Besides mitochondrial respiration and ROS amounts, levels of glucose, amino acid and free fatty acid are altered in LSCs [191,198,201]. Therefore, ex vivo expansion of LSCs requires specific conditions to allow biomarker discovery, drug development, identification of resistance mechanisms and combination treatments.

## 6. Major Challenges Culturing LT-HSCs

Maintaining and expanding LT/ST-HSCs ex vivo are required for curative transplantation therapies and to allow the study of molecular mechanisms [202,203]. The long-term goal is to further optimize application methods for clinical hematopoietic stem cell transplantation (HSCT). 

The effects of cell culture stress on HSCs induces several changes including loss of polarization, accumulation of reactive oxygen species, endoplasmic reticulum stress, genotoxic stress, replicative stress, disturbed protein homeostasis and ultimately loss of HSC functions [204,205,206,207,208,209,210,211]. As a result of these cell intrinsic changes occurring during ex vivo culture, the number of LT/ST-HSCs declines over time accompanied by an increase of myeloid potential [206,207,211]. To reduce environmental stress and preserve stem cell functions, enhanced proteostasis, a dynamic maintenance of proteome integrity, is particularly important in cultured HSCs [207]. Defining culture conditions favoring expansion of LT/ST-HSCs while maintaining their fitness, still represent a major hurdle.

## 7. Maintaining Quiescent LT-HSCs 

The BM has limited oxygen supply; the most quiescent LT-HSCs reside in a “hypoxic niche” where blood perfusion and oxygen tension are low [212,213,214,215]. In vitro hypoxic cultures with 1–3% oxygen enhance LT/ST-HSC expansion and subsequent engraftment [216,217,218,219]. Under normoxia (20% O_2_), the maintenance of self-renewal requires the presence of high cytokine concentrations. Still, normoxia favors differentiation over self-renewal [220].

Kobayashi et al. defined a minimal set of factors that mimic the physiological conditions in the BM microenvironment and maintain LT-HSCs in a quiescent, but still engraftable state for 1 month. Murine LT-HSCs need low concentrations of cytokines (3 ng/mL SCF, 0.1 ng/mL TPO), hypoxia (1% O_2_) and 4% bovine serum albumin (BSA). Supplementing fatty-acids in an albumin-bound form is crucial to avoid intrinsic fatty acid synthesis, which is triggered by hypoxia and low cytokine concentrations [220]. Intrinsic fatty acid synthesis would interfere with HSC survival [221]. Under low cytokine, hypoxia, 4% BSA conditions, differentiation is suppressed and more than 60% of cells remain functional CD150^+^ CD48^−^ LT-HSCs. A gradual decrease of HSC markers including CD150 and EVI1 was only observed after a month of culture. Human LT-HSCs were also maintained under comparable hypoxic conditions in 4% BSA with low cytokines, supplemented with fatty acids and cholesterol. ~90% of the cells exhibited a CD34^+^ CD38^−^ marker phenotype and 40% a CD90^+^ CD45RA^−^ phenotype, reflecting minimal differentiation [220].

Retinoic acid (RA) signaling is high in dormant LT-HSCs compared to ST-HSCs and MPPs [9]. Retinoic acid is produced by two sequential oxidation steps from dietary vitamin A (retinol). The biological active derivative ATRA signals through the retinoic acid receptor (RAR) and the retinoid X receptor (RXR) families [222,223]. The ATRA restrains c-MYC expression, and inhibition of MYC activity partially mimics the preservation of dormancy by ATRA. Treatment with ATRA, retinol or MYC inhibitor retains LT-HSC quiescence ex vivo in serum free, cytokine supplemented (SCF, TPO, FLT3L) media by downregulating G_2_M checkpoints, E2F targets, ROS species and c-MYC targets compared to untreated cells [224].

Recently, purified single mouse (CD45^+^ EPCR^+^ CD48^−^ CD150^+^ SCA-1^high^) and human (CD34^+^ CD38^−^ CD90^+^ CD45RA^−^ CD19^−^ CD49f^+^) LT-HSCs were maintained in a hibernated (hibHSC), non-proliferative state under minimal cytokine conditions (only interleukin-11 (IL-11)) over a 7 days period [225,226,227]. Large proportions of hibHSCs survived without dividing and retained their functional properties, as determined by single-cell transplantation. Stress response pathways together with glycolysis, fatty acid biosynthesis, cAMP and mTOR signaling pathways were upregulated in hibHSCs, most presumably because of nutrient withdrawal and limited cytokine availability [225]. The development of a “quiescent LT-HSC ex vivo system” will open an avenue to study steady-state LT-HSC properties and effects of targeted manipulation.

## 8. Expansion Techniques to Retain LT/ST-HSC Phenotype Ex Vivo

### 8.1. 2D Methods

#### 8.1.1. Suspension Culture of Murine LT/ST-HSCs

Ex vivo culture approaches try to mimic physiological conditions in the BM and provide growth factors and cytokines to maintain quiescence or induce proliferation. Early attempts did not include any supporting BM cells but relied on media supplements like cytokines and growth factors (Figure 2). 

Later, in the 1990s, 5-fluorouracil (5-FU) treated mice were used as a source for collecting and culturing LT/ST-HSCs. They were kept in 20% fetal calf serum (FCS) (or without serum), 1% BSA, SCF, FLT3/FLK-2 ligand (FLT3L) and IL-11 containing media [228]. Frequently, IL-11 is replaced by IL-6 or IL-12 [229]. Further supplements included ITS-X (insulin, transferrin, selenium, ethanolamine) or low-density lipoproteins [229,230,231,232]. Under these conditions, the LT/ST-HSCs maintained their ability to reconstitute lethally irradiated recipient mice for up to three weeks but lost it upon addition of IL-3 and/or IL-1 to the culture medium [228,232,233,234]. In contrast, addition of TPO to mouse BM cells enhanced the number of LT/ST-HSCs and increased the efficiency of BM reconstitution [235]. 

The advanced knowledge about HSC self-renewal and its regulation by niche components allowed for the development of novel ex vivo expansion approaches using different combinations of supplements [236,237,238]. The BM niche cells (e.g., ECs) stimulate self-renewal of LT/ST-HSCs by inducing NOTCH signaling [151,239]. Using the engineered NOTCH ligand Delta1^ext-IgG^, mouse LSK cells were successfully cultured in 20% FBS, SCF, FLT3L, IL-6 and IL-11 supplemented media up to 42 days investigated [240,241]. The immobilized Delta1^ext-IgG^ (composed of the extracellular domain of Delta1 joined to a Fc part of human immunoglobulin G1) accelerates the expansion of LSK*s* and the rate of T-cell reconstitution after transplantation [241,242]. 

Ieyashu et al. showed that interleukin-1α (IL-1α) and hemopexin (HPX) in serum-free, but BSA-containing medium supports the maintenance of LT/ST-HSCs [231]. The heme-binding plasma glycoprotein HPX is expressed on NMSCs in the BM niche and prevents heme-mediated oxidative stress and dampens intracellular ROS levels [243]. The use of SCF and TPO together with IL-1α and HPX provides a highly reproducible ex vivo mouse LT/ST-HSC expansion culture system [231]. 

One of the most frequently used media to enrich LT/ST-HSCs was developed by the group of Lodish. It includes serum-free medium with SCF, TPO, FGF-1, IGF-2, and heparin, resulting in an 8-fold increase of mouse LT-HSCs in three weeks of culture [145,230]. Addition of angiopoietin-like proteins (ANGPTLs: ANGPTL2, ANGPTL3) to the mixture of SCF, TPO, IGF-2 and FGF-1 revealed a roughly 50-fold increase in numbers of repopulating mouse LT-HSCs [230,244]. Although previous protocols used similar media reagents, Lodish’s specific combinations favored proliferation of LT-HSCs over ST-HSCs and prevented them from being outcompeted during long-term culturing.

Another critical feature for successful maintenance of LT/ST-HSCs ex vivo is the use of appropriate BSA, which is a component of most protocols containing fetal bovine serum (FBS). The quality and composition of FBS varies between batches, which leads to differences in culture conditions and affects self-renewal [231,245]. In general, differences in serum and BSA concentrations modulate the bio-availability of cytokines and growth factors based on binding to serum albumin [246]. To standardize culture conditions, BSA was replaced by polyvinyl alcohol (PVA), a synthetic amphiphilic polymer, which stabilizes cytokines. In the presence of PVA, 100 ng/mL TPO and 10 ng/mL SCF are considered optimal for murine LT/ST-HSC culture [226]. This represents a significant improvement and enables long term culture (1- to 2-month) with an up to 900-fold expansion of functional lin^−^ c-KIT^+^ SCA-1^+^ CD150^+^ CD34^−^ LT-HSCs [145,226,227,228,232,247,248]. 

#### 8.1.2. Suspension Culture of Human LT/ST-HSCs

Human cord blood CB-derived hematopoietic stem cells (CB-HSCs) are an important source for HSC transplantations. Their numbers are low in vivo, which requires expanding CB-HSCs ex vivo while preserving their stemness properties for effective application in transplantation and gene therapy. Several promising protocols for serum-free cultivation of human LT/ST-HSCs using combinations of cytokines or small molecules have been described (Figure 2).

Commonly used cytokines for expansion of CD34^+^ HSCs include SCF, TPO, FLT3L, granulocyte colony-stimulating factor (G-CSF), IL-6 IL-3 and FGF-1 [249,250,251]. Using a cytokine cocktail (SCF, TPO, FLT3L and FGF-1) supplemented with insulin-like growth factor-binding protein 1/2 (IGFBP1/2) and ANGPTL5, increased the number of human CD34^+^ CD38^−^ CD90^+^ CD133^+^ CB stem cells. These cells repopulate NOD-SCID mice with a ∼20-fold higher efficiency than non-cultured HSCs [249,251].

Developmental regulators such as NOTCH ligand Delta-1, pleiotrophin, StemReginin-1, UM171, resveratrol, nicotinamide and valproic acid (VPA) were reported to further enhance CD34^+^ HSPCs expansion over 50 fold [250,252].

The FDA approved small molecular weight compounds StemReginin 1 (SR-1) or UM171 are used in addition to cytokines (SCF, TPO, FLT3L and IL-6) to expand human CD34^+^ HSCs ex vivo [253,254,255]. The SR-1 antagonizes the aryl hydrocarbon receptor (AHR), which regulates hematopoiesis through regulation of HES-1, c-MYC, C/EBP, PU.1, β-catenin and CXCR4 [256]. The *Ahr* knockout mice have increased numbers of HSCs with a higher proliferative rate and accumulation of plasmacytoid dendritic cells (pDCs) [256,257]. In line, antagonizing AHR via SR-1 ex vivo enhanced the frequency of CD34^+^ HSCs and induced differentiation of myeloid mDCs and pDCs [255]. In a clinical trial SR–1 has induced a 330-fold expansion of CD34^+^ cells and resulted in fast engraftment of neutrophils and platelets in patients. Neutrophil recovery response has been viewed as a surrogate marker of host immunity [258]. The AHR is also antagonized by Resveratrol, a naturally occurring polyphenol. Resveratrol binds receptors involved in HSC activity including AHR and integrin αvβ3 [259,260]. Addition of Resveratrol to a cytokine-containing (SCF, TPO, FLT3-L, IL-6) medium represents a robust ex vivo method for the expansion of functional CD34^+^ CB HSCs. The UM171 also inhibits LSD1 (H3K4me1/2 demethylase) and HDAC1/2 (e.g., H3K27ac deacetylase), leading to the re-establishment of H3K4me2 and H3K27ac epigenetic marks, which normally rapidly decrease in human LT/ST-HSCs ex vivo [261,262,263]. Of note, when UM171 is combined with SR-1 and cytokines (SCF, TPO, FLT3L, IL-6) the efficiency of expansion is further increased [263].

As a suppressor of SIRT1 deacytelase, nicotinamide inhibits differentiation and enhances expansion of CD34^+^ CB-HSCs [264]. The SIRT1 deacetylates and thereby deactivates p53 protein [265]. Proof for SIRT1´s ability to maintain stemness potential comes from a phase I clinical trial. The surrogate marker, median neutrophil recovery rate, was significantly increased in individuals, who had received nicotinamide treated CD34^+^ CB-HSCs [266].

A screen of CD34^+^ CB-HSCs identified histone deacetylase inhibitor (HDACI) VPA as a promising candidate for LT-HSC expansion. Adding VPA to SCF, TPO, FLT3L and IL-3 in serum free media enhanced expressions of CD90, c-KIT, CXCR4 and integrin α6 (CD49f), increased activation of p53 and reduced ROS levels. The VPA induced HSC expansion by reprogramming CD34^+^ CD90^−^ cells to CD34^+^ CD90^+^ HSCs, accompanied by increased proliferation. The VPA-expanded peripheral blood (PB) cells and BM HSCs established unbiased multilineage human hematopoietic-cell chimerism in NSG mice at 16 weeks post-transplantation [267,268]. 

The major side effect of allogeneic transplantations is the development of graft-versus-host disease (GvHD). Compared to BM HSCs, the transplantation of CB-derived CD34^+^ cells with up to two human leukocyte antigen mismatches revealed a lower risk of GvHD [269]. Due to the low numbers of CB-CD34^+^ cells from a single donor cord, the hematopoietic and immunological recovery of the recipients may be delayed, causing higher infection rates and transplant-related mortality. In the clinics, SR-1, Notch-ligand, UM171 and nicotinamide-based methods have been associated with improved neutrophil recovery early after transplantation, reducing side effects of HSCT [258,266,270]. In spite of major developments in ex vivo expansion of CB-HSCs, the efficiency of their long-term engraftment is still inadequate [271]. To overcome this limitation, double CB transplantations, with nonexpanded CB-HSCs of a second donor, were used to engraft. In this case, a higher risk of GvHD was reported [272]. Thus, enhancing the availability of functional CD34^+^ CB-HSCs would give an excellent opportunity to improve therapeutic applications. 

#### 8.1.3. Suspension Culture of Human LSCs

Several patient-derived AML and CML cell lines are available. They are easy to culture but acquire multiple cytogenetic aberrations upon prolonged culture. This aspect needs to be considered when comparing experiments from different laboratories that may significantly differ [273]. In contrast to primary AML cells, which are genetically and functionally heterogenous (consisting of LSCs and differentiated cells), cell lines are homogeneous, favoring proliferation of the most aggressive clones [274,275,276,277]. Culture systems for primary AML samples that preserve clonal heterogeneity are required to mimic the situation in patients.

To selectively culture CD34^+^ HSPCs from CML/AML samples, a feeder cell-free and serum-free liquid culture system containing FLT3L, SCF, IL-3, IL-6, and TPO has been established. The outcome is highly patient-dependent and shows a great variability [276,278].

The AML specimens share the common feature of high AHR activation in vitro, which provokes differentiation. The AHR agonist SR-1 and other small molecular weight compounds (UM171 and its derivative UM729), counteract differentiation. No stromal co-culture is required but multiple cytokines are necessary, like BIT (BSA, insulin, transferrin), SCF, FLT3L, IL-3 and G-CSF [279,280]. It is currently unclear how the pyrimido-indole UM729 enhances CD34^+^ cell expansion which is AHR independent [280].

Inhibiting GSK-3 and mTORC1 also maintains self-renewing capacity of hematopoietic cells from healthy donors or AML patients [281]. The GSK3 inhibitors (GSK3i) prevent β-catenin degradation and activate WNT target genes, which is essential for long-term HSC self-renewal. The activation of mTORC1 drives HSC proliferation and differentiation, leading to HSC exhaustion [281]. As GSK3-inhibition activates mTORC1 a combined inhibition of GSK3i and mTORC1 is required to maintain self-renewing abilities [282].

### 8.2. 2.5D Methods

#### 8.2.1. Co-Culturing LT/ST-HSCs

The balance between quiescence and activation of LT/ST-HSCs is tightly regulated by the BM microenvironment [90]. The absence of a BM niche leads to a gradual loss of the LT-HSC status and induces differentiation towards lineage-committed progenitors. Co-culture options were developed to mimic the BM microenvironment (reviewed in [283], Figure 2). 

“2.5D co-culture” methods use supporting stromal or endothelial cells to simulate the BM niche. LT/ST-HSCs or LSCs grow on top of adherent cells, either in direct contact (contact culture) or separated by filters (trans-well culture). Trans-well cultures circumvent the need to separate HSC cells from the supporting cells, but are less efficient than cultures that allow for direct stroma contact [284,285]. The MSCs are most commonly used as they express high levels of HSC-supporting factors and significantly improve engraftment [286,287].

Primary MSCs downregulate niche factors (*Scf*, *Cxcl12* and *Vcam1*) upon culture and their ability to maintain HSCs declines over time. Nakahara et al. identified five transcription factors (*Klf7*, *Ostf1*, *Xbp1*, *Irf3* and *Irf7*) that restored HSC niche function in cultured MSCs. Overexpression of these factors revitalized MSCs (rMSCs). The rMSCs expanded cells showed a seven-fold higher efficiency in expansion of functional LT-HSCs in a setting where lineage-depleted mouse BM cells or CD34^+^ CBs were co-cultured in the presence of SCF and TPO [288].

Aside from MSCs, ECs serve as a supporting cell layer. The BM-derived ECs secrete self-renewal supporting angiocrine growth factors, such as VEGF-A and NOTCH ligands [289]. Long-term maintenance of primary ECs involves loss of their angiogenic properties. To preserve them and initiate immortalization, E4orf1 has been introduced. E4orf1 is an adenoviral E4 gene product which confers long-term survival through tonic phosphorylation of AKT [290]. Immortalization of ECs by E4orf1 allows long-term cultures and efficiently supports mouse LT-HSCs when in direct contact in serum-free, SCF supplemented co-culture [289]. The support is mainly provided by the initiation of Notch signaling. In analogy, human fetal liver (FL) sinusoidal ECs engineered and immortalized by E4orf1 (hFLSECs-E4orf1) are used for long-term culture of CD34^+^ CB cells. The hFLSECs-E4orf1 cells also provide activation of NOTCH signaling, mimic the vascular niche and prevent LT-HSC exhaustion [291].

#### 8.2.2. Co-Culturing LSCs

Primary AML cells differentiate and/or undergo apoptosis in culture indicating that most AML cells depend on signals from the microenvironment [280,292,293,294,295]. Co-culture with BM stromal cells expands AML LICs, but frequently selects for a specific subpopulation [296,297,298]. Immortalized mouse BM mesenchymal or endothelial stromal cell lines, such as MS-5, FBMD-1, OP9, HS-5, HS-27 or HUVEC are used as supporting cells for primary AML cultures [114,296,297,299]. The mesenchymal nestin^+^ MS-5 murine BM stromal cell line efficiently maintains functional, chemo-resistant human LSCs ex vivo over 3 weeks [297,298]. The MS5 cells secrete CXCL12, ANGPT1, MCP-1 and HGF; MS5/LIC cocultures were further supplemented with IL-3, G-CSF and TPO and kept at 3% O_2_, which provides a niche-like milieu [300]. The hypoxia signaling pathway triggers LIC maintenance in vivo [301]. 

Primary MSCs isolated from AML patients have an impaired ability to support normal HSPCs, but an enhanced ability to maintain LSCs compared to MSCs from healthy donors [292,302,303]. Leukemic BM niche MSCs have different morphology and growth rate, altered osteogenic or adipogenic differentiation capacity and changed methylation signatures [292,302,303,304,305,306,307]. These co-culture conditions are labor-intensive and require careful standardization. Among the critical factors is the cell density of the feeder cells, which must be in a cell cycle arrested state. Passage restrictions of the feeder cells are required to avoid stromal cell line exhaustion and ensure comparable experimental conditions [297,308]. 

### 8.3. 3D Methods 

#### 8.3.1. 3D Culture of LT/ST-HSCs 

The 3D culture methods attempt to mimic the spatial structure of the BM microenvironment by providing cell-to-cell or cell-to-biomimetic matrix contacts (Figure 2). Cell spheroids or aggregates are grown on a matrix, or in a scaffold-free suspension. Commonly used scaffold/matrix materials include natural polymers, such as alginate, Matrigel™ (basement membrane matrix), agarose and bacterial nanocellulose, hyaluronic acid or synthetic-based polymer materials [296,309,310]. Polyethylene glycol (PEG), PVA, poly (lactic-co-glycolic acid) (PLGA), poly (lactic acid) (PLA) and poly (ε-caprolactone) (PCL) are common materials used to form synthetic scaffolds hydrogels [311]. 

To generate scaffold-free spheroids, different cell types (e.g., MSCs and LT-/ST-HSCs) are cultured on hanging drops, microplates, non-adhesive surfaces or via a forced floating method (magnetic levitation or agitation-based approaches) to initiate aggregation and to avoid attachment to a culture dish [309,312,313,314,315,316]. Spheroids have some advantages compared to 2.5D cultures; (i) higher levels of hematopoietic niche factors provided by MSCs, (ii) the maintenance of the cell shape and (iii) improved signaling by cell-cell contacts increased ex vivo LT-/ST-HSC expansion compared to 2D and 2.5D methods [312,317].

The LT-/ST-HSCs cells can also be encapsulated into a natural or synthetic polymer solution that is cross-linked to form a hydrogel. Hydrogels are biocompatible, retain large amounts of water and provide excellent permeability [318,319]. Hydrogels are mixed with ECM proteins such as fibronectin, collagen, laminin or glycosaminoglycans to allow attachment of LT-/ST-HSCs [309,318]. Cytokines, small molecules or other factors can be incorporated depending on the aim of the research project [320].

Compared to a PEG scaffold, zwitterionic hydrogels are super-hydrophilic and are more resistant to non-specific protein binding [319,321]. Bai et al. set up a zwitterionic hydrogel system to ex vivo culture BM and CB LT-/ST-HSCs, using a metalloproteinase-cleavable zwitterionic peptide to reversibly crosslink the gels. The LT-/ST-HSCs-secreted metalloproteinases gradually cleave peptide crosslinker, allowing cells to actively shape their environment [320]. This improved cell migration, cell-cell contacts and stemness of the HSCs; and proved to be an efficient method to expand functional CD34^+^ LT-HSCs based on reduced differentiation, diminished reactive oxygen species (ROS) production and a low metabolic rate [319,320,321].

A disadvantage of encapsulation is the homogenous matrix, unlike the porous sponge-like structure of the BM. Although cells have full contact to the hydrogel/ECM matrix, a direct cell-cell contact may be missing. To circumvent this problem, porous scaffolds were generated (e.g., by salt leaching, freeze-drying or 3D printing), where MSCs and CD34^+^ CB-HSCs can form close cell-cell contacts, improving ex vivo expansion [322,323,324]. Alternatively, Wharton’s jelly, can be employed as a 3D matrix. Wharton’s jelly is a mucoid connective tissue surrounding the umbilical cord vessels to confer a mechanical protection in the womb. Decellularized Wharton’s jelly matrix (DWJM) serves as ECM scaffold and is mixed with primary human MSCs to maintain ex vivo CD34^+^ CB-HSCs cells. The DWJM shares many components of the BM ECM including collagens, fibronectin, tenascin-C, hyaluronic acid and numerous sulfated glycosaminoglycans possessing many unique biochemical characteristics [291,325,326]. 

Currently, self-sustained 3D applications like small-scale microfluidic devices and large-scale bioreactors are developed. Bioreactor is an engineered system that allows culturing large amounts of CD34^+^ CB-HSCs through automated control over medium supply, waste removal and agitation [327]. Microfluidic devices mimic the vascular niche by continuously supplying nutrients and oxygen, while generating gradients (e.g., oxygen, calcium ions, cytokines and small molecules) [328,329]. Bone marrow-on-a-chip is an even more complex microfluidic system, where the colonization of the 3D matrix by niche cells takes place in the BM in vivo [330,331]. 

#### 8.3.2. 3D Culture of LSCs

In an effort to model the BM microenvironment for ex vivo leukemia studies, stiff and porous 3D scaffolds have been used [332,333,334,335,336,337]. Leukemia research is inclined to find strategies to disrupt the protective effect of the niche cells to improve therapeutic strategies. Bray et al. co-seeded ECs and MSCs with primary AML cells in matrix metalloproteinase–sensitive PEG-heparin hydrogels, supplemented with growth factors (VEGF, FGF-2, stromal cell-derived factor 1 (SCD1)). The protective effect of leukemic-vascular interactions increased chemoresistance of cancer cells in 3D compared to 2D [334].

The first bioreactor system was recently developed to maintain malignant CD34^+^ cells from AML and myeloproliferative neoplasm (MPN) patients for up to 3 weeks. The system contained human osteoblastic BM niches, engineered by MSCs in 3D porous scaffolds under perfusion flow [113].

The 3D methods represent the future to expand LT/ST-HSCs in large quantities for transplantations, but are less suitable for individual research-purposes, as they are technically challenging and labor-intensive. The use of 3D techniques is limited when it comes to high-throughput multi-well experiments, such as drug-screening, but represents the best choice for any validation experiments.

## 9. Immortalized Hematopoietic Stem/Progenitor Cell Lines 

**Figure 3:**
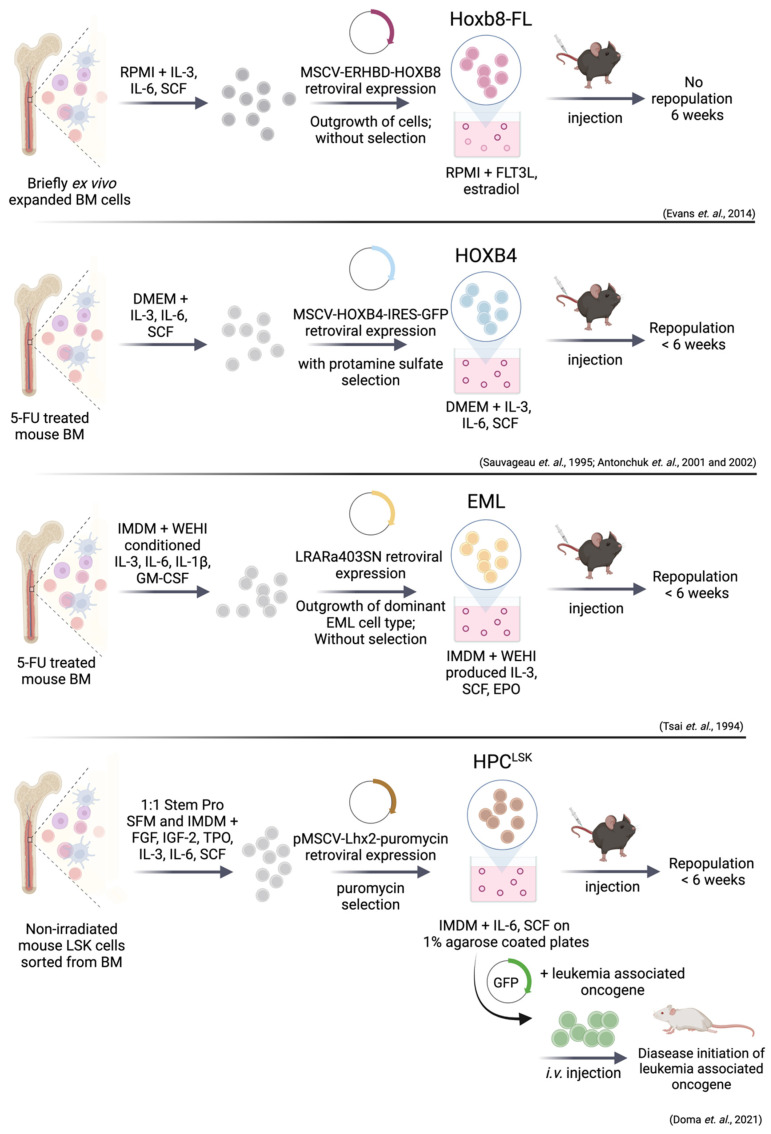
Simplified visualization of unique cell system methods for combined in vitro and in vivo studies of genetically modified HSPCs. Retroviral overexpression of Hoxb8-FL, HOXB4, RARα403 and Lhx2 enforces HSC self-renewal and proliferation. Addition of cytokines is a key factor for long-term culture of HSPCs. The HPC^LSK^ cell lines can be transformed with oncogenes including BCR/ABL^p210^, FLT3-ITD;NRAS^G12D^ and MLL/AF9. One critical step for generation of a HSPC cell line includes the engraftment for more than 6 weeks. Hoxb8-FL cells do not fulfill this requirement of reconstitution capacity.

Genetic manipulation of self-renewal pathways by transgene overexpression may provide a suitable method for HSPC expansion and the establishment of cell lines. Successful HSPC immortalization was achieved by overexpression of embryonic developmental genes including *HOXB,* *RARα* and *Lhx2* to enforce cell renewal and arrest cell differentiation [338,339,340] (Figure 3).

Bulk- or LSK-sorted mouse BM cells were isolated and cultured in serum, growth factor and cytokine supplemented media. Dividing cells were immortalized through retroviral expression of *HOXB4* [146,341], *HOXB8* [342], truncated *RARα*, (RARα403) [343] or *Lhx2* [339] and resulted in rapid and extensive ex vivo expansion of HSPC populations for more than 9 weeks (Figure 3). Exogenous growth factors are still required to induce proliferation. This process raised long-term expanded, progenitor cell lines that remained multipotent as demonstrated by their ability to fully repopulate lympho-myeloid lineages in primary and secondary recipients. All transplanted mice remained healthy and without manifestations of hematopoietic disorders after one year of observation [146,339,341,342,343].

### 9.1. Immortalization via HoxB8 and HoxB4

The HOX proteins are a family of evolutionary conserved transcription factors. In mammals, 39 *HOX* genes are organized into four distinct clusters: *HOXA*, *HOXB*, *HOXC* and *HOXD* [344]. A considerable amount of data link *HOXA* and *HOXB* genes to cell renewal and the arrest of cell differentiation [338,345]. These functions were exploited experimentally to establish stably growing, homogenous hematopoietic progenitor cell lines through retroviral expression of *HOX* genes (*HOXB4* and *HOXB8*). Expression confers growth advantage but does not elicit leukemic potential [146,341,342]. 

Downregulation of *Prdm16* might be involved in preventing leukemia in HOXB4 overexpressing LT/ST-HSCs transplanted mice [346]. The transcription regulator PRDM16 is associated with AML, causes oncogenic fate conversion from megakaryocyte-erythroid progenitors (MEPs) to LSCs, by interacting with super enhancers and activating myeloid master regulators, including PU.1 [347]. The PRDM16 was markedly repressed by HOXB4, but upregulated by HOXA9 and HOXA10 [346]. This may explain why HOXB4 and probably also HOXB8 lacks the leukemogenic potential seen with other oncogenic HOX factors such as HOXA9 and HOXA10.

In Hoxb8–FL cell lines, the hormone binding domain of the estrogen receptor (*Erhbd*) is fused to the coding sequence of *Hoxb8,* leading to the activation of Hox genes by estrogen. Estrogen withdrawal provokes differentiation into dendritic cells (DCs). Besides estrogen, growth and survival of these cells strictly depends on FLT3L [342] (Figure 3). Hoxb8–FL cells have both myeloid and lymphoid potential but lack any megakaryocyte and erythroid capacities and closely resemble MPP4 cells (lin^−^ SCA-1^+^ cKIT^+^ CD48^+^ CD150^−^ CD34^+^ CD135^+^) [6,342,348]. One month after transplantation, Hoxb8–FL cells are no longer detectable in the BM, but are still present in spleen, peripheral blood, and thymus. This observation suggests a compromised capacity to self-renew. The Hoxb8–FL–derived T–cells reached merely about 10–30% of physiological T–cell numbers in the thymus, but were absent in the periphery [342]. Within the four HSPC lines discussed here, Hoxb8–FL shoes the least multipotency.

The Hox factor HOXB4 is a key regulator of LT-HSCs self-renewal but its enforced expression allows differentiation when transplanted [341,349]. The factor *HOXB4* drives proliferation by upregulating AP-1 complexes with subsequent enhanced cyclin D1 levels [350]. Combination of overexpression of *HOXB4* with deletion of the HOX cofactor *Pbx1* (pre-B-cell leukemia transcription factor 1) or expression of *NUP98-HOXB4* fusion protein further enhances ex vivo expansion of LT-/ST-HSCs [351]. Both approaches (*HOXB4/Pbx1* KO and *NUP98-HOXB4)* reconstituted myeloid and lymphoid populations in vivo without inducing leukemia [351,352,353,354]. Recent findings indicate that HOXB4 may also reprogram induced pluripotent stem cells (iPSCs) cells into long-term repopulating HSCs, opening new avenues for human therapeutic possibilities [355,356].

### 9.2. EML (Erythroid, Myeloid, and Lymphocytic) Cell Line

The EML cells originally emerged as an in vitro model to study self-renewal and lineage commitment. The EML cells express a truncated, dominant-negative form of the human RAR (RARα403) [343,357] (Figure 3). The RARα403 outcompetes the endogenous RAR in the formation of biologically active RAR/RXR complexes, leading to c-MYC upregulation and proliferation. As mentioned above, ectopic RA signaling maintains LT/ST-HSC quiescence, while inhibition leads to stem and progenitor proliferation [224,343].

The EML cells generate large numbers of B-lymphoid and erythroid progenitors at the expense of progenitors for the neutrophil and macrophage lineages. This may be overridden by a combination of IL-3 and high concentration of RA, which increases the number of myeloid progenitors [343]. One advantage of this cell system is the ability for in vitro T-cell differentiation (upon co-culture with murine OP9-DL1 stromal cells in the presence of SCF, IL-7, and FLT3). OP9-DL1 cells express delta-like-1, a NOTCH ligand which drives human and murine HSPCs into T-cells in vitro [358,359].

The main limitation of EML cells cultivated with SCF is their functional heterogeneity containing CD34/SCA-1^low^ and ^high^ populations. Each subpopulation expresses a distinct pattern of HSPC markers and transcription factors, different multilineage differentiation potential and proliferation kinetics [357]. CD34/SCA-1^high^ EML cells exhibit low GATA1 and high PU.1 level, which predisposes them to the myeloid lineage. In contrast, CD34/SCA-1^low^, GATA1 high, PU.1 low cells are erythroid-prone [360]. Besides GATA1, other erythroid genes including *α-* and *β-hemoglobin, Epor*, *Eraf* (erythroid associated factor) and mast cell proteases are expressed at high levels in the CD34/SCA-1^low^ EML cells [361]. The EML cells serve as a model for cell intrinsic and extrinsic pathways that regulate plasticity among multipotent hematopoietic cells.

Ectopic expression of HOXB4 in EML cells allows HSC self-renewal through upregulation of stemness-related genes, such as *Laptm4b*, *Gp49a*, *Sox4*, and *CD34.* HOXB4 downregulates erythroid and B cell lineage-specific genes to keep cells in a primitive state [362]. This cell system extends our knowledge on molecular and functional properties of HSPCs but lacks the ability of long-term cultivation without differentiation. 

### 9.3. Immortalization via Lhx2—HPC^LSK^ Cell Lines

Retroviral transduction of the LIM-homeodomain (LIM-HD) transcription factor *Lhx2* was used to generate multipotent HPC^LSK^ cell lines [339,363,364] (Figure 3). The LHX2 has a critical role in hematopoiesis and *Lhx2*-null embryos die in utero with severe anemia [365,366]. The critical role of LHX2 in hematopoiesis was underlined by forced expression in embryonic stem (ES) cells, which resulted in the outgrowth of multipotent SCF-dependent progenitor cells [363]. The LHX2 upregulates hematopoietic self-renewal genes and inhibits differentiation of mouse ES derived LSK cells [367]. 

The SCF/IL-6 dependent HPC^LSK^ cells are kept on agarose-coated plates to prevent adherence-induced myeloid differentiation. The HPC^LSK^ cells efficiently home to the BM, blood, spleen, and thymus and can differentiate into myeloid and lymphoid lineages in vitro and in vivo. Numbers of HPC^LSKs^ -derived myeloid and lymphoid progenitors in the BM and differentiated blood cells are comparable to BM-injected mice [339]. Transcriptome analysis of HPC^LSKs^ showed a ST-HSC/MPP1 (lin^−^ SCA-1^+^ cKIT^+^ CD48^−^ CD150^−^ CD34^+^ CD135^−^) signature, which corresponds to the earliest proliferating stem/progenitor cells despite expression of CD48 and CD150 [6,339,348]. The absence of any cell feeder layer or extensive amounts of cytokines makes them a robust and low-cost model system that guarantees long-term multipotency.

### 9.4. Leukemic Stem Cell Lines Using the HPC^LSK^ System

The HPC^LSK^ cells can be genetically modified e.g., by retroviral transduction to generate LSCs lines harboring hematopoietic stem cell oncogenes including BCR/ABL, MLL-AF9 or FLT3-ITD;NRAS^G12D^ [141,339,368,369] (Figure 3). The SCF/IL-6 dependency can be overcome by HPC^LSK^ BCR/ABL^p210+^ cell lines, which grow cytokine-independently. 

Injection of transformed HPC^LSK^-LSCs (BCR/ABL^p210+^, MLL-AF9, FLT3/NRAS^G12D^) lines in immunocompromised mice induced myeloid leukemia. All diseased animals displayed elevated WBC counts, blast-like cells in the blood and suffered from splenomegaly [339]. The HPC^LSK^ BCR/ABL lines demonstrated similar transcriptional and phospho-signaling signatures compared to BCR/ABL CML patients [368]. Another advantage of the HPC^LSK^ system is the ability to rapidly generate cells lines from transgenic mouse models. The HPC^LSKs^ lines have been generated from *Cdk6^−/−^*, *Stat5a^−/−^* and *Stat5b^−/−^* transgenic mice [141,339]. When HPC^LSK^ BCR/ABL^p210+^ *Cdk6^−/−^* cells were transplanted, loss of CDK6 was associated with a reduced incidence of leukemia, mimicking the effects of published primary BM transplantation assays [175,339]. These data verified the potential of the HPC^LSK^ system to generate diverse leukemia models. 

Recently, a unique role for STAT5B in driving self-renewal of HSCs/LSCs was described, additionally using the HPC^LSK^ system to underline the in vivo/ex vivo results. The factor STAT5B, but not STAT5A, is predominantly present in the nucleus of HPC^LSK^ cells stimulated with cytokines (TPO, EPO, GM-CSF) or transformed cells [141]. These assays underline the broad application of the HPC^LSK^ cell lines for functional assays that require high cell numbers. These findings support the relevance of HPC^LSK^ cell system that represents a unique tool to compare LSCs to non-transformed HPC^LSK^ cells in vitro and in vivo, which can be adapted for high-scale preclinical compound screening.

## 10. Conclusions

We here summarize the current knowledge on methods for ex vivo cultivating primary LT/ST-HSCs, LSCs, and generating in vitro HSPCs cell lines. In general, enhanced self-renewal ability comes at the expense of differentiation. Self-renewal is orchestrated by BM niche signals, including cytokines, cell-ECM, and cell-cell interactions while LSCs are additionally affected by the aberrant expression of oncogenic drivers.

Several approaches are available to promote self-renewal including co-cultivation on a stromal feeder layer or embedding in a BM-mimicking matrix, in the presence of cytokines. Alternatively, self-renewal may also be maintained via genetic modifications.

Murine and human primary HSCs cultures serve distinct purposes. Human LT/ST-HSC expansion aims primarily at improving conditions for transplantation settings in personalized medicine. Most preclinical studies use murine LT/ST-HSCs and ex vivo expanded primary LSCs as they are a versatile system to address research questions. Murine systems are instrumental in understanding the pathways regulating HSC quiescence and allow new medical perspectives through drug screening and biomarker discovery approaches. 

Despite advances, culturing, isolating, and maintaining primary stem cells are still challenging. It is a labor-intensive process resulting in low numbers of cells, which prevents the conduction of high throughput techniques. Murine HPC^LSK^ cells and HPC^LSKs^ derived LSCs provide a solution, as they can be expanded indefinitely and facilitate basic and mechanistic studies. The HPC^LSK^ lines thus represent a quick, reliable, and reproducible tool to study hematopoietic malignancies and large-scale drug sensitivity/resistant assays. 

Despite encouraging results and novel in vitro/ex vivo assays, strategies to selectively target quiescent LSCs, the key drivers of relapse, are still elusive. Their identification will pave the way towards the development of new treatment strategies for AML and CML patients.

## Figures and Tables

**Table 2:** LSC-specific cell surface markers present both in AML and CML cells.

Human LSC Surface Markers					
AML and CML Markers	Alternative Name	Refs.	AML and CML Markers	Alternative Name	Refs.
CD25	IL-2Rα chain	[71,72,73]	CD33	Siglec-3	[72,74,75,76]
CD45RA	CD45, also known as protein tyrosine phosphatase, receptor (PTPRC)	[77,78]	CD93	C-type lectin-like domain (CTLD) containing glycoprotein	[79,80,81]
CD9	Motility Related Protein-1 (MRP-1) Tetraspanin-29 (TSPAN29)	[72,82,83]	CD123	Interleukin 3 receptor subunit α (IL-3R α)	[72]
CD371	C-type lectin domain family 12 member A (CLEC12A); CLL-1 antigen	[72,84]	IL1RAP	Interleukin 1 receptor accessory protein	[85,86]
CD69	C-Type Lectin Domain Family 2, Member C (CLEC2C) Activation Inducer Molecule (AIM)	[87,88,89]	CD36	Thrombospondin Receptor Platelet Collagen Receptor Glycoprotein IIIb (GP3B)	[88,89]
CD43	Leukosialin	[75]	CD44	Pgp-1, multidrug resistance protein 1 (MDR1)	[75]
CD45	Leukocyte common antigen (LCA)	[75]	CD157	Bone marrow stromal cell antigen 1 (BST1)	[75]
